# Impact of increasing the proportion of healthier foods available on energy purchased in worksite cafeterias: A stepped wedge randomized controlled pilot trial

**DOI:** 10.1016/j.appet.2018.11.013

**Published:** 2019-02-01

**Authors:** Rachel Pechey, Emma Cartwright, Mark Pilling, Gareth J. Hollands, Milica Vasiljevic, Susan A. Jebb, Theresa M. Marteau

**Affiliations:** a Behaviour and Health Research Unit, Institute of Public Health, University of Cambridge, Cambridge, UK; b Nuffield Department of Primary Care Health Sciences, University of Oxford, Oxford, UK

**Keywords:** Food, Availability, Energy, Cafeterias, RCT, Pilot

## Abstract

Increasing the proportion of healthier foods available could encourage healthier consumption, but evidence to date is limited in scope and quality. The current study aimed to: (a) examine the feasibility and acceptability of intervening to change product availability in worksite cafeterias; and (b) estimate the impact on energy purchased of increasing the proportion of healthier (i.e. lower energy) cooked meals, snacks, cold drinks and sandwiches. Six English worksite cafeterias increased the proportion of healthier foods available, aiming to keep the total number of options constant, in a stepped wedge randomized controlled pilot trial conducted between January and May 2017. The intervention was generally successfully implemented and acceptable to clientele. Generalized linear mixed models showed a reduction of 6.9% (95%CI: -11.7%, −1.7%, p = 0.044) in energy (kcal) purchased from targeted food categories across all sites. However, impact varied across sites, with energy purchased from targeted categories significantly reduced in two sites (−10.7% (95%CI: -18.1% to −2.6%, p = 0.046); −18.4% (95%CI: -26.9% to −8.8%, p = 0.013)), while no significant differences were seen in the other four sites. Overall, increasing the proportion of healthier options available in worksite cafeterias seems a promising intervention to reduce energy purchased but contextual effects merit further study.

## 1 Introduction

Patterns of unhealthy behavior, including excessive energy intake, are key contributors to non-communicable diseases, which currently cause the majority of premature deaths worldwide (Forouzanfar et al., 2015; Wang et al., 2016). Cues within small-scale physical environments – e.g. within shops, restaurants or bars – can increase selection and consumption of high energy foods often without awareness, also known as “nudging” (Hollands, Marteau, & Fletcher, 2016; Thaler & Sunstein, 2008; Wood, 2017). Considerable attention has recently been paid to the potential impact of altering these physical micro-environments in order to encourage healthier choices (Hollands, Bignardi, et al., 2017; Marteau, Ogilvie, Roland, Suhrcke, & Kelly, 2011; Thaler & Sunstein, 2008). This approach has its foundations in dual process theories (Hofmann, Friese, & Strack, 2009; Strack & Deutsch, 2004), leading to the hypothesis that changing environments is more effective than information-based interventions, as it does not necessarily rely on individuals’ cognitive resources (Marteau, Hollands, & Fletcher, 2012; Wood, 2017). As such, these interventions may be more equitable (McGill et al., 2015), given cognitive resources may be depleted by both deprivation in childhood and current financial burdens (Mani, Mullainathan, Shafir, & Zhao, 2013; Moffitt et al., 2011; Raver, Blair, & Willoughby, 2013).

One setting worthy of particular attention for public health nutrition interventions is the workplace (Black, 2008; Wanjek, 2005; World Health Organisation, 2013), where employees are estimated to consume a sizable proportion of their daily energy intake. Interventions that lead to even small reductions in energy consumed at work could help to offset the energy excess which underpins population-level weight gain. Most adults consume more energy than recommended – by 200 kcal per day on average in the UK, where self-reported energy intakes are lower than in the US (Public Health England, 2018; Vernarelli, Mitchell, Rolls, & Hartman, 2018) – and it has been estimated that just an extra 16 kcal per day could equate to a weight gain of 6.2 kg over a 10-year period (the median weight gain from 1999 to 2009 for UK 20-40 year-olds) (Calorie Reduction Expert Group, 2011).

One possible intervention is to alter the availability of higher vs. lower energy foods in workplace cafeterias. At a basic level, the presence or absence of a product inevitably fixes the possible options. Increasing the options for a particular type of product (e.g. healthier food items) within a set of choices may also increase the probability that a product within this set meets a consumer's purchase goals (e.g. appeal, price, etc.) (Chernev, 2012). Beyond this, exposure to a product could affect a range of processes that are broadly part of reward systems (Schultz, 2016) – for example, increasing the salience of the product and the attention directed towards it, as well as the “mere exposure” effect (Zajonc, 2001) whereby repeated exposure increases the appeal of an object. The range of products might also affect selection through implying a social norm (Reno, Cialdini, & Kallgren, 1993), with increased availability of healthier options having the potential to imply a new norm, and thereby alter behavior. Exploring these largely untested possible mechanisms – once there is more evidence of the intervention effect – has the potential to optimize intervention effects as well as developing the science of behavior and behavior change.

Although the underlying mechanisms remain uncertain, altering the availability of foods is one of the top three interventions suggested in the McKinsey Global Institute report (2014) as having the highest likely impact on obesity across the population. The evidence underpinning this intervention, however, remains limited in both scope and quantity. A recent review of interventions in vending machines (including in worksites, as well as schools, universities, and hospitals) identified seven studies that increased the availability of healthier foods, five of which found that sales of these foods increased with no loss of overall sales volume (Grech & Allman-Farinelli, 2015). A review of worksite interventions (Allan, Querstret, Banas, & de Bruin, 2017) identified two studies focused on increasing the availability of healthier foods as a single intervention strategy, both showing introducing fruit baskets led to increased fruit intake (Alinia et al., 2010; Backman, Gonzaga, Sugerman, Francis, & Cook, 2011). A further eight studies in this review altered availability – including those implementing a broader suite of changes to product numbers or ranges – as part of multicomponent interventions (Allan et al., 2017). Similarly, studies in military cafeterias have altered availability but again alongside other interventions (Bingham et al., 2012; Crombie et al., 2013), finding an impact on the healthiness of food selected but unable to isolate the independent effect of altering availability. A Cochrane review of availability interventions in all settings is currently underway (Hollands, Carter, et al., 2017).

However, to our knowledge, there have been no studies to date that isolate the impact of increasing the availability of healthier options across a broad range of items. In the current study, healthier options are defined as lower in energy – selected as a pragmatic outcome, while acknowledging this is only one dimension of healthier diets. The current study aims to:

1. Assess the feasibility and acceptability of conducting a trial altering the availability of products in worksite cafeterias;
2. Explore the impact on energy purchased of increasing the proportion of lower energy options, while aiming to keep constant the number of options available.

## 2 Methods

### 2.1 Design

**Fig. 1:**
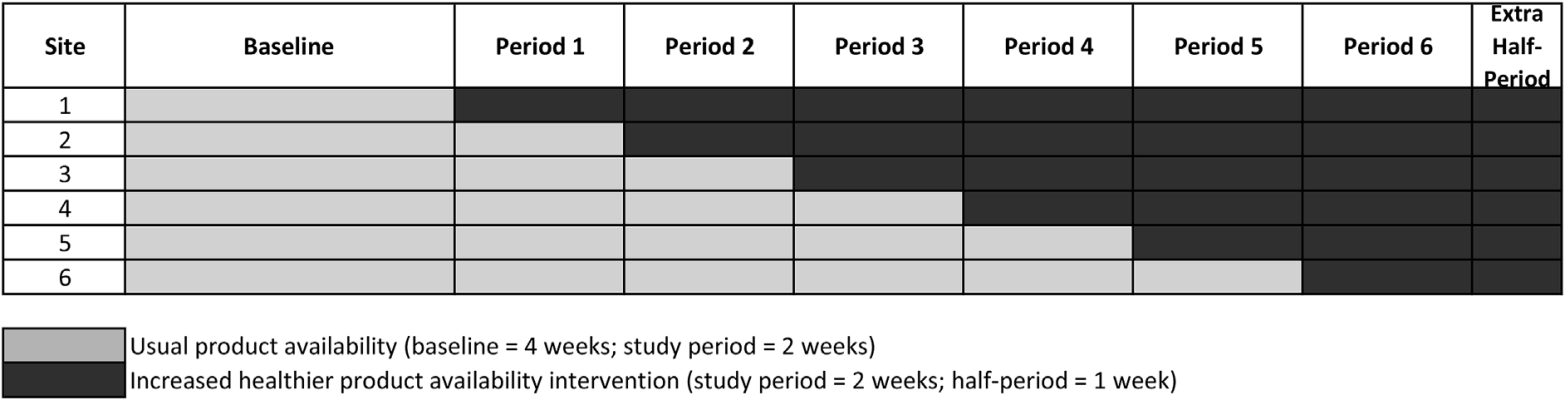
Stepped wedge design employed in the study.

A stepped wedge randomized controlled trial design was used, conducted between January and May 2017 (trial registration: http://www.isrctn.com/ISRCTN52923504; study protocol: Vasiljevic et al. (2017)). A 4-week baseline period undertaken by all sites, after which one site was randomized to implement the intervention every two weeks (see Fig. 1). An additional intervention period of one week was undertaken by all sites at the end of the trial, such that the minimum intervention period for any site was 3 weeks. Once randomized to the intervention, intervention changes were maintained until the end of the study (i.e. intervention length per site varied between 3 and 13 weeks). Simple randomization to intervention start dates was conducted by the research unit's Statistician using computer-generated random numbers (obtained using R statistical software, version 3.3.1). Allocation was not concealed: the research team enrolled the worksites and assigned them to the random sequence for intervention implementation. Ethical approval for the study was obtained from the University of Cambridge Psychology Research Ethics Committee (Ref: Pre.2016.035).

### 2.2 Sites

**Fig. 2:**
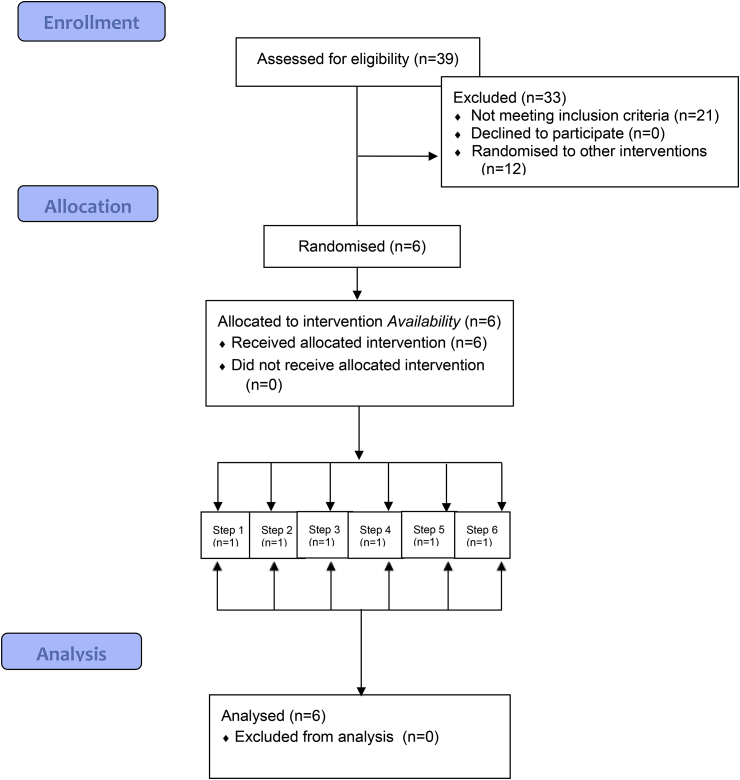
CONSORT flow diagram.

Six worksite cafeterias were recruited, through IGD (Institute of Grocery Distribution: https://www.igd.com/) (see Fig. 2: CONSORT flow diagram). The decision to include six sites for this pilot study was determined for pragmatic reasons (i.e. maximizing sample size, given available resources). IGD invited managers of worksites that: (a) were based in England, (b) had approximately 350 or more employees, and (c) could provide at least weekly sales data on individual items and the energy (kcal) content of items sold. Sites were selected to represent a mix of office-based and depot/manufacturing sites. Three sites (Sites 3, 4 and 5) form part of the same company campus, with a shared manager. As a result, one of the sites volunteered and subsequently recruited (Site 3) had fewer than 350 employees.

### 2.3 Intervention

The intervention comprised increasing the number of healthier options available while decreasing the number of less healthy options available by the same extent (i.e. changing the proportion of healthier options without decreasing the overall number of choices). Healthier and less healthy options were operationalized by their energy content; while this does not encompass the full picture with regard to healthier diets, targeting lower vs. higher energy options was selected as a pragmatic outcome to examine the general effectiveness of interventions targeting healthier food availability. The intervention focused on increasing the availability of healthier cooked meals, sandwiches, snacks (subdivided into sweet and savory) and cold drinks, given these were the areas items could be swapped most readily.

#### 2.3.1 Cooked meals

Healthier cooked meals (excluding breakfast) were defined as having under 300 kcal for a meal component typically served with an additional potato or rice side (e.g. a fish cake), or under 500 kcal for a complete meal (e.g. burritos). This was based on Public Health England (Change 4 Life) suggesting a 400–600 – 600 kcal split between Breakfast – Lunch – Dinner. The 500 kcal limit here allows 100 kcal for vegetables and/or drinks.

Sites were asked to limit less healthy meal options to one per day during the intervention. If sites had rolling menus, the research team suggested specific meal options to swap, starting with highest energy options. Sites were also asked to limit less healthy potato or rice sides (defined as sides with added fat, e.g. chips, roast potatoes or mash) to one option per day.

#### 2.3.2 Sandwiches

Healthier sandwiches (or equivalents, e.g., wraps, panini, baguettes, bagels) were defined as those under 350 kcal. This cutoff allowed for a snack and a drink to be included as part of the recommended energy allowance for a lunch, following the same guidance as for cooked meals. The aim was to increase the proportion of healthier sandwiches offered to 50%.

#### 2.3.3 Snacks and cold drinks

We applied cutoffs to define whether options were healthier or less healthy:

- Savory snacks: under 120 kcal per pack (e.g. Popchips 23g)
- Sweet snacks: under 150 kcal per pack (e.g. Nakd bar 30g)
- Cold drinks: under 50 kcal per pack (e.g. zero or light varieties)

These thresholds were based on the 400 kcal remaining in the 2000 kcal per day for women following the Public Health England suggestions, described above. This would allow for two snacks and two drinks daily. The cutoff for savory snacks was lowered to 120 kcal to reflect the generally lower kcal per pack values in this category (mean 170 kcal based on items identified at recruitment, compared to 215 kcal per pack for sweet snacks), to ensure that meaningful changes were made within both sweet and savory snack categories.

For each of these three categories (savory snacks, sweet snacks and cold drinks), we increased the number of healthier options to 50% based on the items identified as being available on recruitment. If sites already had close to or more than half of options classed as healthier in a particular category, the proportion was instead increased to two-thirds.

#### 2.3.4 Selection of options to swap

Items selected to be removed from sites were those with the highest energy content per pack, apart from cold drinks, for which those with the highest energy values per 100 ml were selected for removal to ensure a range of healthier options in both cans and bottles. Exceptions to this rule were snack items that consisted of fruit, nuts and seeds without added sugar or salt, and 100% fruit juice, which were classed as healthier. If a site was unwilling to swap a particular item (e.g. fish and chips on Fridays, or both cans and bottles of Coca-Cola), the next highest energy item was selected instead. Healthier replacements were agreed with each site, matched to the category of the original item.

### 2.4 Procedure

A list of products available was obtained from each site upon recruitment, and an initial site visit conducted to assess feasibility. The research team identified targets for intervention, and agreed these with the catering teams. Sites were asked to keep available products as consistent as possible during the study. Cafeteria customers were not informed of the study.

Each site underwent an initial 4-week baseline period. Sites then implemented the intervention following a stepped-wedge design, i.e. one site starting the intervention at each fortnightly step. During the intervention period, sites were asked to make all the changes agreed upon recruitment. They were asked to position replacements in the same location, and with as close as possible to the same number of packs, as the removed product, and to restock these as usual. Replacement products were priced at their recommended retail price or using the catering providers’ normal pricing guidance.

Daily sales data were obtained from the till records of each site. Data on the energy content of each item sold were provided by each site.

### 2.5 Measures

#### 2.5.1 Feasibility

The feasibility assessments, as detailed in the protocol (Vasiljevic et al., 2017), are summarized here:

1. *Feasibility of recruiting and retaining sites*: assessed by recruitment and drop-out rates.
2. *Feasibility of implementing the intervention*: assessed after initial visits to worksite cafeterias by the research team, in discussions and formal interviews with worksite managers and catering teams, and through examination of the sites' sales data.
3. *Acceptability of the intervention*: measured by surveying worksite cafeteria customers, and qualitative interviews with worksite managers or catering managers.
4. *Compliance with the study protocol*: assessed during compliance visits conducted during the first week following intervention implementation for each worksite, and photographs sent weekly thereafter.

#### 2.5.2 Intervention impact

*Primary outcome:* Total energy (kcal) purchased per day from intervention categories.

*Secondary outcomes:* Total energy (kcal) purchased per day from all categories.

Total revenue (GBP) per day from all categories.

Covariates:

1. The number of items purchased from non-intervention categories, included as a proxy for site busyness
2. Day of the week (dummy variables), given within-week fluctuations in sales
3. The number of days pre-/post-intervention, to allow for trends over time
4. Price increases implemented in three sites during the study period (indicated using a common dummy variable as the price changes were consistent between sites)
5. Free lunches provided to employees (Site 3 supplied packed lunches on three days and held a retirement buffet on another day; indicated using a dummy variable)

##### 2.5.2.1 Changes from published protocol

Several changes have been made since the publication of the protocol for this study (Vasiljevic et al., 2017):

- The primary outcome has been limited to energy purchased from targeted food categories, given that it was only appropriate and possible to intervene in certain categories. This was agreed prior to any inspection of the data, and changed in the trial registration at that time (http://www.isrctn.com/ISRCTN52923504
- One site could not provide data on the number of daily transactions. We instead use amount purchased from non-intervention categories to control for site busyness.
- To avoid over-specifying the model, analyses do not include site-level covariates.
- Issues with price increases and supply of free lunches were identified after commencement of the study and publication of the protocol paper, so were added retrospectively as covariates.

### 2.6 Analysis

#### 2.6.1 Feasibility and acceptability

Recruitment and dropout rates, along with semi-structured interviews and researcher assessments, were used to assess study feasibility. Feedback from worksite cafeteria patrons was summarized using descriptive statistics to complement the results from the qualitative interviews in assessing study acceptability.

#### 2.6.2 Intervention impact

*Data cleaning and imputation of missing data:* The research team matched energy information provided by sites to sales data, following these procedures:

- Multiple items recorded under the same till button: The median energy value of items recorded under the till button was taken as the energy content of these items.
- Missing energy data:
  o For cooked meals, energy content was estimated by taking the mean of three recipes matching the meal's description. These recipes were obtained from recipe banks of the catering providers across the enrolled sites, or from food recipe websites that provided calorie information, e.g. BBC Good Food, if information was unavailable from the catering providers. When the description provided was too generic to match to a recipe (for example, sales of “Special Hot Filled Roll” on days where no corresponding option was listed on the menu), the mean for that type of cooked option (e.g. “Hot rolls”; “Main Course”) in that site pre- or post-intervention was used.
  o Items for which no information was available across the study period (e.g. self-serve salad boxes) were left as missing.
- Missing sales data: For Site 1, data for 17th-26th Feb were recorded incorrectly, and could not be obtained later – these data were treated as missing.

*Analysis:* Generalized linear mixed models were fitted in R.3.3.3 to estimate the potential impact of the intervention, with the unit of analysis being worksite cafeteria per day. The daily energy purchased was logged in analyses due to heteroscedasticity of the residuals in untransformed models. P-values were calculated using the robust Kenward-Roger adjustment, in order to minimize the potential for bias given the small sample size, which could otherwise inflate Type-I error rates (Kenward & Roger, 1997; McNeish, 2017).

Analyses examined the impact of altering product availability (modelled using a dummy variable for intervention periods) on the log of total energy (kcal) purchased per day from targeted food categories, controlling for the number of items purchased from non-intervention categories, day of the week, number of days pre-or post-intervention, price increases (at Sites 3, 4, and 5) and site-supplied free lunches (at Site 3), with random effects for worksite. Weekends and public holidays were excluded.

To examine the impact in each site separately, six separate dummy variables indicating the intervention period in each site replaced the overall intervention dummy variable in a follow-up analysis.

*Secondary outcome:* Using the same analysis, and controlling for the same covariates, the impact of the intervention on daily energy (kcal) purchased from all products with energy information was also examined.

Exploratory analyses looking at the impact of the intervention on the revenue (GBP per day) at each site were also conducted, controlling for the covariates listed above, but with transactions replacing the number of items purchased from non-intervention categories, given the potential for the latter variable to affect revenue beyond accounting for site busyness. This analysis only included Sites 2–6, as transactions data were missing for Site 1.

##### 2.6.2.1 Sensitivity analyses

*Use of median energy value for items recorded using the same till button:* Some items with varying energy content were sold under the same till button (e.g. ‘San Pellegrino Can’; between 119 and 139 kcal per can). We took the median energy value to represent such items. As it is possible that purchasing may in fact follow a healthier or less healthy bias, we also ran the analyses using the 25th percentile and 75th percentile energy values of the different items sold under each of these till buttons, to assess the impact of this on results.

*Delayed implementation at one site:* At Site 4 only the removal of targeted pre-packaged items (two cold drink options) occurred on the scheduled implementation date; their replacements went on sale three days later (due to delays obtaining these from suppliers), and the cooked meals were changed six days later (due to staff shortages). Given the limited changes on the implementation date, we also ran analyses for this site with the intervention coded as starting from when the intervention was fully implemented (six days later).

## 3 Results

Six sites were recruited between September and December 2016, and participated in the study from January to May 2017. The sites represented a range of business activities, reflected in the predominant occupational group employed at each site (see Table 1).

**Table 1:** Site characteristics.

	Site					
	1	2	3	4	5	6
Type of site	Depot	Office	Office	Manufac-turing	Office	Manufac-turing
No of employees	960	2176	165	749	816	334
Percentage of employees that are full-time	95.7	96.0	97.0	92.3	82.2	99.7
Mean employee age^a^	40.1	36.5	40.9	44.3	38.0	34.9
Percentage of employees that are female	21.9	49.7	40.0	17.4	55.1	16.2
Predominant occupational group^b^	D&E	C1&C2	C1&C2	D&E	A&B	C1&C2
Cost of cooked meal (£)	1.17	3.90	2.45^c^	2.45^c^	2.45^c^	2.52^d^
Cost of pre-packaged item pre-intervention (£)	0.41	1.02	0.63	0.64	0.73	0.99
Cost of pre-packaged item post-intervention (£)	0.44	1.06	0.68	0.71	0.74	1.07
Usual number of daily cooked meal options offered by site	3	4	3	4	5	3
Number of different (healthier) pre-packaged items sold at baseline e^,^^f^	89 (28)	150 (42)	33 (3)	25 (8)	67 (21)	96 (24)
Percentage of energy (kcal) purchased from targeted food categories from cooked meals^g^	47.0%	86.7%	83.6%	84.3%	75.6%	57.2%

a Reported in age bands, and estimated using the mean age value for employees in each age band.

b A&B: Higher and intermediate managerial, administrative and professional occupations; C1&C2: Supervisory, clerical and junior managerial, administrative and professional occupations; D&E: Semi-skilled and unskilled manual occupations.

c Changed to £2.65 for last 5 weeks of study.

d Mean value as no standard price.

e Proxy for number of items available pre-intervention; where multiple products are sold under one till button, all known possible items are taken as being sold.

f Study criteria for healthier pre-packaged items: Cold drinks: under 50 kcal per pack; Savory snacks: under 120 kcal per pack; Sweet snacks: under 150 kcal per pack.

g Based on pre-intervention sales.

Table 2 presents the intervention characteristics by site, showing that the range of items altered, extent of changes and fidelity of intervention implementation varied by site. For most categories in most sites, the proportion of healthier options requested was 50%. Exceptions were the cold drink and savory snack categories at Site 3, and the cold drink category at Site 5, where 50% were already classed as healthier, so for these categories the proportion of healthier options requested was two-thirds of items.

**Table 2:** Intervention characteristics (*Pre-Int: Pre-Intervention; Post-Int: Post-Intervention*).

		Site					
		1	2	3	4	5	6
Proportion of food items for which energy content is available		76.1	77.2	87.8	82.6	81.1	93.9
Proportion of food items in targeted food categories		75.2	69.4	71.8	66.1	67.4	83.1
Proportion of cooked meals targeted in the intervention		12.5^a^	0	15.3	41.7	40.0	25.0
Proportion of days where cooked meal offering fails study criteria^b^	Pre-Int.	31.8	–	41.5	100	100	63.8
	Post-Int.	33.3	–	0	11.4^d^	0	0
Mean energy (kcal) per cooked meal sold (s.d.)^c^	Pre-Int.	337 (101)	627 (301)	475 (166)	589 (182)	579(206)	388 (160)
	Post-Int.	342 (107)	637 (320)	403 (136)	424 (163)	352 (113)	321 (115)
Number of pre-packaged items identified as targets for intervention		15	29	11	2	17	12
Number of pre-packaged items added (removed) in the intervention		8 (12)	29 (29)	11^d^ (11)	2^d^ (2)	15 (17)	11^d^ (12)
Mean energy (kcal) per pack for pre-packaged items (s.d.)	Pre-Int.	154 (150)	130 (84)	128 (76)	92 (85)	135 (80)	145 (80)
	Post-Int.	135 (95)	95 (75)	91 (77)	79 (79)	111 (73)	115 (77)
Mean daily energy (kcal) purchased from targeted food categories (s.d.)	Pre-Int.	201,371 (24,709)	178,325 (32,790)	29, 444^e^ (10,000)	47,948 (7461)	114,921 (21,577)	85,638 (14,544)
	Post-Int.	207,392 (30,085)	160,342 (31,044)	27,437 (4634)	43,242 (8488)	85,420 (9224)	81,524 (12,612)

a No fixed menus, so this is the proportion of cooked meals that would need to be changed to meet study criteria pre-intervention.

b Study criteria for healthier cooked meal offering: Only one less healthy cooked meal offered daily (cutoffs: 300 kcal for a meal served with a side; 500 kcal for a complete meal).

c Cooked meal energy totals include sides for some sites but not others, depending on the energy information provided by sites and/or how they record sales of cooked meals and sides, so comparison across sites may not be appropriate.

d Delays in being introduced: Site 3: 3 items by 2 weeks; Site 4 (cooked meals): all by 6 days; Site 4 (pre-packaged): 2 items by 3 days; Site 6: 1 item by 1 week.

e If the days the site provided free lunches are excluded: 32,068 (6199).

Table 2 highlights that the average reduction in energy content post-intervention was larger for a cooked meal option than a pre-packaged item, which, given the high proportion of sales comprised by cooked meals at most sites (Table 1), suggests that changes here may be more influential than changes to the pre-packaged offering.

### 3.1 Feasibility

All six sites approached agreed to participate, and none dropped out during the study period. In terms of implementation, some categories proved more amenable to intervention than others. Five of the sites agreed to intervene on cooked meals (Site 2 declined), although in one of these sites (Site 1) one-third of days did not meet the study intervention criteria during the intervention period, and comparison of the proportion of cooked meals meeting the healthier criteria pre- and post-intervention (Table 2) suggests little change from pre-intervention. For the other four sites, the adherence to the cooked meal changes was very high, other than a delay in implementation at Site 4. It should be noted that Sites 1 and 2 did not follow a rolling menu, making it harder to plan the intervention for cooked meals. In contrast, individual meal substitutions were agreed in advance for Sites 3–6.

All of the sites intervened on pre-packaged items - all six on cold drinks, and the five sites offering snack options intervened on these (Site 4 only offered a very limited range: 5 cold drink options and no snacks). Site 6 was the only site to intervene on sandwiches. There was relatively high adherence to the requested swaps for pre-packaged items (with the exception of introducing lower energy options in Site 1 due to issues with sourcing items).

In terms of specific items to swap, three sites (Sites 1, 2 & 3) wanted to keep one of their highest energy cold drinks and Site 1 did not wish to swap two of their highest energy savory snacks (equating to 6% of pre-packaged targets shifting to the next highest energy option). Four sites (Sites 3–6) did not want to remove fish and chips on Fridays (3% of targeted cooked meals), due to concerns about possible negative reactions from customers.

Discussions with cafeteria managers also highlighted several occasions where delays to pre-packaged item replacements arriving meant that intervention implementation was partially delayed. In addition, the process of agreeing and implementing proposed changes was complicated by the involvement of multiple parties – including worksite company management, catering team, catering company management, suppliers and the research team (see Box). Nevertheless, intervention implementation was generally successful for four out of the five sites intervening on cooked meals, and for all sites for pre-packaged items.

Box **Managers' reflections on the study**

**Overall response of customers**

“People were saying that we were dictating to them what they could eat, dictating to them how they run their own diets” *Site 1*.

“I think, there wasn't necessarily really a positive or a negative reaction to it, it was just people accepting of the catering offering for that day and making a choice to actually what was available” *Sites 3-5*.

“They're set in their ways especially with energy drinks and other stuff like that so changing a few things we thought there was going to be a bit more uproar than there actually was but it was, it actually went quite, pleasantly well considering what we thought” *Site 6*.

Variation in responses by employee demographic characteristics.

“I think the demographic of the site is interesting because the people that were complaining are the people that are full on, on the shop floor, picking, doing lots of manual labor so their work ethic, lifestyle choices is a lot different from say a transport driver who sits in a lorry cab for between nine and 12 h a day driving a vehicle around the country” *Site 1*.

“I think possibly the demographic of the office is more biased towards health so I think they responded well to [the intervention]” *Site 2*.

**Acceptance of specific changes**

“We had lots of complaints about returning the original full fat drinks if you want to call them such a thing. Particularly the energy drinks, the energy drinks is probably one of our biggest sellers, and I can only imagine the sugar content on them is massive but it's what the customer demand was throughout the whole thing” *Site 1*.

“The offering of some alternatives in terms of snack items I think were well received by the employees because actually we're currently still retaining those” *Sites 3-5*.

**Process**

“I think the organization of it before it started was slightly challenging and that was, I suppose, getting commitment from [the chef] and the team on what we were doing and I think for [the chef], he probably would have appreciated almost being told what to do and I think although it was coming from [worksite company] because [catering company] were part of it as well if they'd given real clear direction to him that this was a priority and that he needed to focus on it I think that might have made things slightly easier, although in the end we still got to making some changes” *Site 2*.

“A number of the proposed elements in terms of snacking items weren't actually part of our typical purchase and therefore there was a process that [the catering company] had to undertake in order to get those onto the deliveries and things like that” *Sites 3-5*.

**Managers’ perceptions**

“I think it was one of those things when you first get told about it you think ‘oh here we go’. When you actually get into it, it actually does open your eyes to a few things that are possibilities and stuff like that with regards to people's attitudes when they're buying” *Site 6*.

Alt-text: Box

### 3.2 Acceptability

The Box highlights some of the comments made by managers in the interviews. The response from customers was mixed across sites, with interviews suggesting that complaints were made about the changes to products at Sites 1 and 4 in particular, while at the other sites customers were described as largely being positive or indifferent to the intervention. Managers identified site demographics as playing a possible role in acceptability, which may be reflected in the perceived lower acceptability at Sites 1 and 4, both predominantly occupational group D&E (i.e. semi-skilled and unskilled manual occupations).

Only 2% of the 5200 potential cafeteria customers responded to the survey asking two questions relating to intervention acceptability. For the question, “How did you feel about products changing?”, 127 cafeteria patrons (across the six sites) responded, of whom 24.4% felt pleased or very pleased, 25.2% felt neither pleased nor displeased, 23.6% felt displeased or very displeased, and 19.7% didn't notice the changes. Of the 117 respondents to the second question, “Would you like to see the changes remain?”, 40.2% answered yes (either “yes, definitely” or “yes, probably”), 37.6% didn't mind, while 22.2% answered no (either “no, probably not” or “no, definitely not”). As such, while most respondents seemed to find the intervention acceptable or were indifferent to its implementation, a sizeable minority (around 22–24%) were unhappy with the changes. Interviews with managers suggested that complaints from customers were largely focused on the removal of favored products from sale (e.g. energy drinks, full sugar Coca-Cola).

### 3.3 Intervention impact

**Fig. 3:**
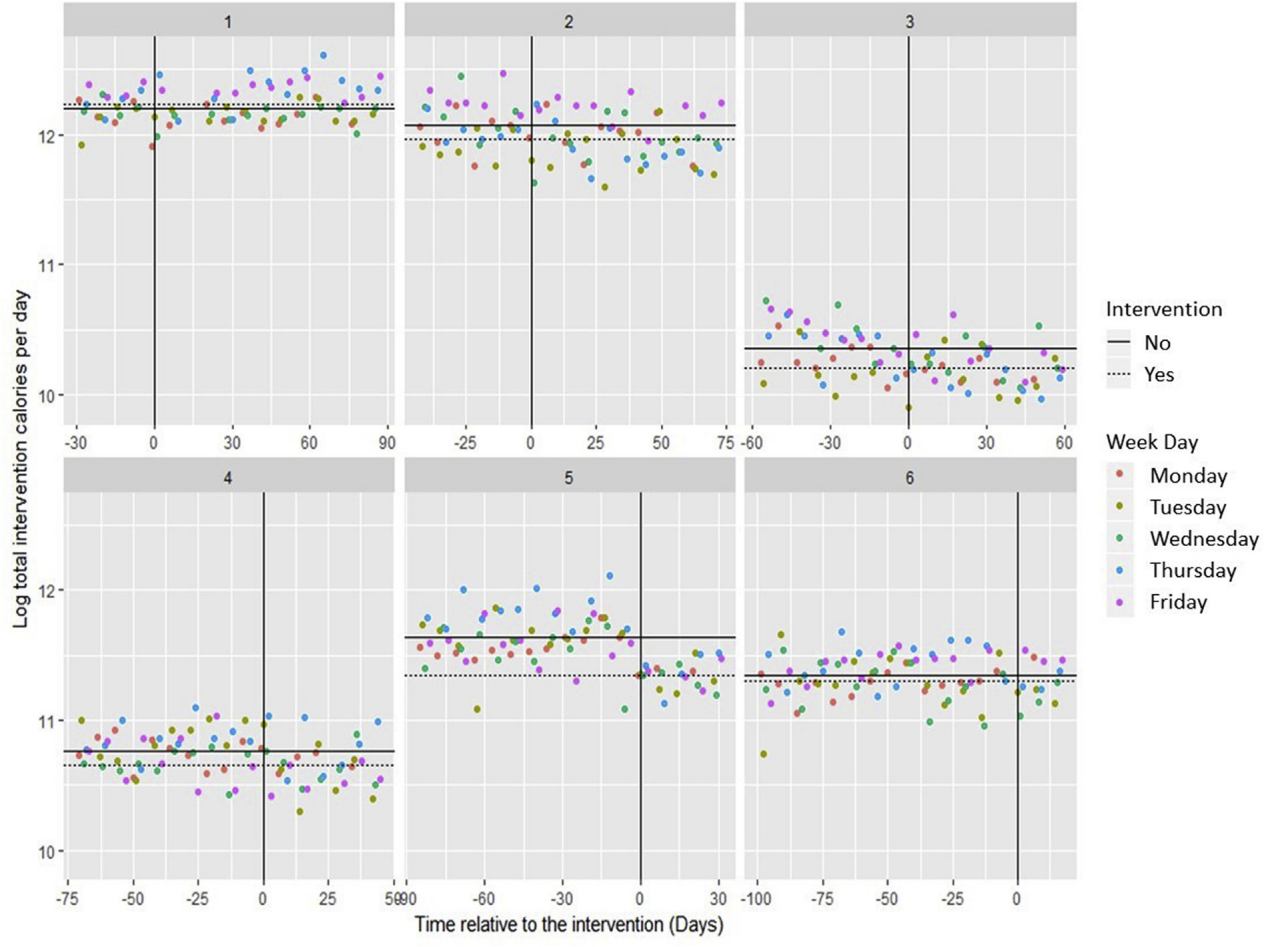
Scatterplots of daily energy purchased (logged) from targeted food categories over time, by site. Solid black lines indicate the mean (on log data) at each site pre-intervention; dotted lines post-intervention. N.B. The plot for Site 3 excludes four days when free lunches were supplied.

The total mean daily energy (kcal) purchased per site in the four weeks of baseline was 108,279 (s.d. 67,005), and in the last three study weeks (when all sites were running the intervention) was 99,316 (s.d. 65,181). Fig. 3 shows the daily energy purchased by site, indicating that in general sites had relatively steady sales pre-intervention, with the suggestion of possible falls in energy purchased post-intervention for Site 5 in particular.

The average effect of the intervention across the six sites was a reduction of 6.9% (−6595 kcal, 95% CI: -11.7% to −1.7%; p = 0.044) in the total daily energy purchased from targeted food categories (see Supporting Information S1 Table for all coefficients from model).

Table 3 shows the results of the analysis by site. (As the outcome was logged in analyses, the coefficients have been back-transformed into percentage change to aid interpretation.) The direction of the effects was consistent with reductions in energy purchased from targeted food categories in five of the six sites, with estimated decreases of between 4% and 18% by site (Site 1 being the exception with an estimated 0.7% increase). In Sites 3 and 5, the intervention significantly reduced energy purchased from targeted food categories, by 10.7% (−3152 kcal, 95% CI: -18.1% to −2.6%; p = 0.046) and 18.4% (−21,095 kcal, 95% CI: -26.9% to −8.8%; p = 0.013) respectively. Differences were not statistically significant in the other four sites (see Supporting Information S2 Table).

**Table 3:** Results of the analysis by site.

Variable		Coefficients (95% CIs)^a^	Percentage change (95% CIs) a	p^a^	Change in energy purchased (kcal)	Mean number of transactions (s.d.)	Change in energy purchased (kcal) per transaction
Availability intervention period	Site 1	0.007(-0.083, 0.099)	0.68 (−8.08, 10.28)	0.889	1374	Unknown	N/A
	Site 2	−0.087 (−0.169, −0.005)	−8.37 (−15.64, −0.47)	0.088	−14,919	989 (87)	−15
	Site 3	**−0.113** (−0.199, −0.028)	−10.70 (−18.12, −2.62)	0.046	−3152	161 (20)	−20
	Site 4	−0.057 (−0.148, 0.035)	−5.50 (−13.87, 3.70)	0.281	−2635	482 (47)	−5
	Site 5	**−0.203** (−0.312, −0.093)	−18.36 (−26.90, −8.81)	0.013	−21,095	847 (76)	−25
	Site 6	−0.043 (−0.145, 0.060)	−4.21 (−13.65, 6.25)	0.450	−3607	230 (24)	−16

Coefficients in bold are significant at p < 0.05.

a As the p-values and CIs presented here have been calculated using different assumptions (it is not possible to calculate 95% CIs that correspond to the more robust Kenward-Roger adjusted p-values), the 95%CIs may cross zero while the p-values are not significant.

Analyses also explored models including random slopes for sites (i.e. allowing each site to have a different effect over time). Model comparisons suggested these provided no improvement over the models presented here, so the simpler model was preferred. These conclusions were also robust to alternative specifications of the time variable (i.e. using days since study start or dummy variables for month, rather than days pre-/post-intervention).

#### 3.3.1 Secondary outcomes

*Total energy purchased:* Results were similar when using total energy purchased from cafeterias rather than energy purchased from targeted food categories (see Supporting Information S3 Table), with an overall reduction of 7.2% (95% CI: -11.2% to −3.0%; p = 0.021) post-intervention. Analysis by sites again suggested significant reductions at Sites 3 and 5 (by 9.5% [95% CI: -15.7% to −2.8%; p = 0.046] and 19.2% [95% CI: -26.1% to −11.6%; p = 0.013], respectively). Analyses suggested reductions in energy purchased in the other four sites (between 0.7% and 7.8%), but these were not statistically significant.

*Revenue:* Analyses suggested that at the five sites where reductions were seen (Sites 2–6), there was no significant difference overall in daily revenue taken by sites post-intervention (taking categorical covariates at their reference values and continuous covariates at their means, daily mean revenue was predicted to be £7.74 higher post-intervention (95% CI: -£10.39 to £26.14; p = 0.454). Analysis by sites suggested no significant differences in revenue at any of the sites (see Supporting Information S4 Table).

#### 3.3.2 Sensitivity analyses

*Estimated energy for items sold under the same till button:* Using either the 25th percentile or 75th percentile energy value rather than the median (i.e. assuming a bias towards healthier or less healthy purchasing) did not substantially change the results, with the exception that if a less healthy pattern of purchasing is assumed (75th percentile), Site 1 also shows a significant reduction in purchasing (by 18.1%, 95% CI: -25.3% to −10.3%; p = 0.007).

*Site 4 implementation delay:* If we take the start of the intervention at Site 4 as the date by which all changes were implemented (as only the removal of pre-packaged items occurred on the scheduled implementation date), the overall effect of the intervention is estimated to be greater (a reduction of 7.4%; 95%CI: -12.2% to −2.3%; p = 0.035), and at Site 4 the size of the reduction (albeit non-significant) rises to 8.2% (95% CI: --16.9% to −1.5%; p = 0.154).

## 4 Discussion

### 4.1 Feasibility and acceptability

The planned intervention of increasing the proportion of lower energy food options available, while aiming to keep the total number of options constant, was implemented according to protocol for the majority of proposed changes. For the pre-packaged items (cold drinks, snacks and sandwiches), all the targeted items were removed at five of the six sites (with 80% of removals made at Site 1), and all the replacements made at three sites (67%, 88% and 92% for Sites 1, 5 and 6, respectively). In terms of cooked meals, all substitutions were made at four of five sites due to make changes (albeit one after a short delay), but implementation at the remaining site [Site 1] was poor (67% of intervention period cooked meals meeting study criteria – similar to pre-intervention levels).

These results suggest that, with the exception of Site 1, fidelity to protocol was reasonably high. At Site 1, many items were grouped under the same till button, and items under the same button often covered a relatively broad range of energy content compared to other sites. This means that the impact of the intervention is more difficult to assess in this site, reflected in the change in estimates for this site in sensitivity analyses examining the impact of multiple item till buttons. It should also be noted that Site 1 did not have a pre-defined menu, and as such specific cooked meal substitutions could not be agreed in advance, but were left to the catering team to decide from a list of recipes, at the start of each week. Similarly, Site 2 also had no pre-defined menus, and declined to intervene on cooked meals. This suggests that implementing this intervention on cooked meals without rolling menus may not be feasible with the intervention set up used in the current study.

In terms of acceptability while, by the study end, most respondents either favored or did not object to the intervention remaining in place, a sizeable minority reported being unhappy with the changes made. Removal of favored pre-packaged products seemed to be a key issue (perhaps as these changes were more overt than changes to cooked meals); these could be substituted back in and another swapped out if necessary to increase intervention acceptability (albeit with the caveat that targeting popular products may have greater impact). These surveys were placed at till points or on tables in cafeterias for customers to complete, and attracted a low response rate. It is possible that customers were not motivated to complete these questionnaires, perhaps because there was low concern about the changes made. Responses are therefore more likely to be biased towards those with stronger feelings about the changes implemented. Higher responses might be achieved in future studies by encouragement by management for survey completion or handing out questionnaires to those using the cafeterias.

### 4.2 Impact

To our knowledge, this is the first study attempting to isolate the impact of intervening on the proportion of lower energy options available across a broad range of food categories in organizational settings. Analyses of the impact of the intervention suggested energy purchased per day across the six worksite cafeterias from targeted food categories was reduced by 6.9% (95% CI: -11.7% to −1.7%), with no significant impact on revenue. The results suggest that it may be possible to make a meaningful reduction in the total energy purchased without affecting revenue by substituting a number of higher energy items for lower energy items (aiming to keep the total number of options available constant). This provides a more robust investigation of implementing changes to product availability than previous studies (which were multicomponent and/or limited in scope), demonstrating the potential for such interventions to be successfully implemented, which may lead to changes in purchasing behavior in worksite cafeterias.

This effect reflects the pattern across worksites (rather than being driven by reductions in one site), with reduced energy purchased being observed in five of the six sites, with significant decreases in two. The exception was Site 1, where we observed lower fidelity to protocol and sales data were of lower quality. While the significant reductions at Sites 3 and 5 were not observed at the other sites, the small sample size limits the interpretation of this result. Differences between sites include the extent of intervention implementation at different sites, the popularity of foods chosen for substitution at each site, and site characteristics. For example, the socioeconomic status of employees may have an impact on their willingness to purchase lower energy substitutes, given socioeconomic patterning in the healthiness of diets (Maguire & Monsivais, 2014; Pechey et al., 2013), and therefore potentially in familiarity with these substitutes. In particular, investigating any potential differential responses by socioeconomic status would be interesting, given the suggestion that environmental changes might be more likely to help address socioeconomic disparities in health-related behavior (McGill et al., 2015). Further studies investigating a larger number of sites or utilizing individual-level data could help to address some of these uncertainties.

#### 4.2.1 Strengths and limitations

This study offers a novel assessment of the feasibility, acceptability and impact of an intervention focused on changing one aspect of the small-scale physical environment (or choice architecture) – increasing the proportion of lower energy food items available. The study introduced lower energy food items (at their usual prices) and removed higher energy options across a range of food categories, while aiming to keep the total number of options constant. This provides a more robust estimate of the potential impact of an availability intervention than previous studies that focused on altering a smaller number of items or were unable to disentangle the specific impact of availability from other elements of multicomponent interventions. The inclusion of multiple worksites, and a relatively long period of implementation add to the robustness of these findings. Finally, analysis of the economic impact of this intervention in terms of revenue provides an indication of the potential cost to organizations of implementing such changes in their worksite cafeterias.

There were, however, several limitations to this study. Firstly, in terms of data recording, in those cases where multiple items were recorded under the same till button assumptions had to be made about the relative levels of purchasing of these items, which varied in energy content (and sensitivity analyses suggested that these assumptions could potentially have impacted on the results for Site 1 in particular). Some sites introduced new till buttons to distinguish healthier/less healthy items for the study period to mitigate this issue, but this may have increased the potential for errors in recording.

A further limitation of data recording is that the outcome measure did not capture the full picture of consumption at the worksites – the purchasing outcome does not reflect food waste or food brought in from elsewhere, chocolate and/or fruit were available free of cost at some sites, and purchasing data from vending machines could not be obtained. While the locations of most of these sites (with the exception of Site 2) were relatively isolated, with limited alternative means of purchasing food beyond the cafeterias, establishing whether compensatory consumption occurs is a key question for future research.

A third limitation relates to the precision of study implementation: given continual small changes to non-intervention product availability during the study period, it was not possible to determine the exact proportion of pre-packaged items available that met the study criteria for healthier options. Moreover, this led to an issue at Site 4 where no higher energy cold drinks were available for a short period (leading to complaints from customers), despite study design calling for higher energy options to always be available for each targeted food category. In addition, sites sometimes did not want to swap items with the highest energy content, due to concerns about customer reactions, which may have reduced the potential impact of the intervention. That said, these limitations are helpful to reflect on to inform future implementation of such an intervention if rolled out to more sites.

Other potential limitations included Sites 3, 4 and 5 all being located on the same campus, making it possible that there was some overlap between customers at these cafeterias, although these workforces are largely separate. Finally, for those sites that used rolling menus, the extent of the intervention on cooked meals varied according to menu week – however, given the limited sample size, it was not possible to model this effect.

#### 4.2.2 Implications for research and policy

The results of this study suggest that intervening to increase the proportion of lower energy options available in organizational settings may be a promising strategy to reduce energy purchased and in turn consumed. Moreover, these results indicate that it may be possible to reduce the energy purchased by workforces in their site cafeterias without impacting revenue. There is a timely need for interventions with the potential to shift behavior at population level, given the current burden of obesity and other diet-related diseases (Forouzanfar et al., 2015; Wang et al., 2016). Interventions targeting aspects of small-scale physical environments, such as product availability, have been advocated (Marteau et al., 2012; McKinsey Global Institute, 2014), but evidence of impact has been largely absent. While the current study shows that it may be possible to shift purchasing to decrease energy purchased (albeit in a small sample), this approach could also be used to target other markers of healthier food consumption, such as saturated fat, sugar or salt intake.

Further research could establish the extent to which the variation in energy reductions by site may be due to different factors, including intervening in different product categories and the socioeconomic status of customers. Moreover, future studies could help elucidate the mechanisms underlying the impact of altering the proportion of healthier food options – e.g. increased visibility, salience and/or product appeal. While larger studies are needed to replicate these results and explore the most effective ways of altering availability, the current study provides a robust signal that this is a promising intervention to consider as part of efforts to reduce consumption of less healthy foods in organizational settings.

## 5 Conclusions

Increasing the proportion of lower energy food options available in worksite cafeterias – while aiming to keep the total number of options constant – may reduce energy purchased, without affecting revenue. While larger studies are warranted to precisely estimate the impact of altering healthier product availability, this intervention could be considered in a wide range of organizational settings to promote healthier food environments. Given that impact varied by site, future research should establish the most effective ways of implementing this promising intervention to ensure that potential benefits to both public health and employee health are maximized.

## Funding

The study is funded by the National Institute for Health Research Policy Research Programme (Policy Research Unit in Behaviour and Health [PR-UN-0409-10109] and IGD (Institute of Grocery Distribution) [RG83425]). RP is supported by a Wellcome Trust Research Fellowship in Society and Ethics [106679/Z/14/Z]. The funders had no role in the study design, data collection, analysis, or interpretation. The research was conducted independently of the funders, and the views expressed in this publication are those of the authors and not necessarily those of the NHS, the National Institute for Health Research, the Department of Health and Social Care or its arm's length bodies, and other Government Departments.

## Declarations of interest

None.

## Data statement

The data are commercially sensitive, provided by the participating worksites on condition that they are not shared beyond the research team.

## Appendix A Supplementary data

s1_V2 s1_V2

s2_V2 s2_V2

s3_V2 s3_V2

s4_V2 s4_V2

Consort_V2 Consort_V2

Data Profile Data Profile
