# Supplementary material for: Impact of increasing the proportion of healthier foods available on energy purchased in worksite cafeterias: A stepped wedge randomized controlled pilot trial
Source: Appetite. 2019 Feb 1;133:286–96. doi: 10.1016/j.appet.2018.11.013 (PMC6335439; doi:10.1016/j.appet.2018.11.013)
Supplement: s1_V2 [file mmc1.pdf]

SUPPORTING INFORMATION

S1 Table. Regression coefficients from all variables in the primary analysis aggregating across sites (impact on logged energy purchased from targeted food categories)

Variable		Coefficients (95% CIs)[1]	Percentage change (95% CIs)[1]	P[1]
Availability intervention period		**-0.071** (-0.124, -0.018)	-6.86 (-11.72, -1.74)	0.044
Number of items sold in non-intervention categories		**0.0004** (0.0003, 0.0006)	0.04 (0.03, 0.06)	0.002
Number of days pre- or post-intervention[2]		-0.00009 (-0.0008, 0.0006)	-0.009 (-0.080, 0.062)	0.816
Day of the week (Ref = Monday)	Tuesday	-0.025 (-0.072, 0.021)	-2.50 (-6.92, 2.13)	0.329
	Wednesday	-0.014 (-0.061, 0.032)	-1.42 (-5.91, 3.30)	0.573
	Thursday	0.062 (0.013, 0.110)	6.40 (1.36, 11.69)	0.049
	Friday	0.049 (-0.003, 0.100)	5.07 (-0.23, 10.65)	0.115
Price increase		**-0.104** (-0.160, -0.048)	-9.86 (-14.83, -4.61)	0.013
Site-supplied free lunches		**-1.77** (-1.93, -1.61)	-83.00 (-85.55, -79.98)	<0.0001
Intercept		**11.10**		<0.0001

[1] As the p-values and CIs presented here have been calculated using different assumptions (it is not possible to calculate 95% CIs that correspond to the more robust Kenward-Roger adjusted p-values), the 95%CIs may cross zero while the p-values are not significant.

[2] Day 0 is the day that the intervention was implemented

Coefficients in bold are significant at $p<0.05$.
