# Supplementary material for: Impact of increasing the proportion of healthier foods available on energy purchased in worksite cafeterias: A stepped wedge randomized controlled pilot trial
Source: Appetite. 2019 Feb 1;133:286–96. doi: 10.1016/j.appet.2018.11.013 (PMC6335439; doi:10.1016/j.appet.2018.11.013)
Supplement: s2_V2 [file mmc2.pdf]

S2 Table. Regression coefficients from all variables in the primary analysis by site (impact on logged energy purchased from targeted food categories)

**Variable**		**Coefficients (95% CIs)**[1]	**Percentage change (95% CIs)** [1]	**p**[1]
Availability intervention period	Site 1	0.007 (-0.083, 0.099)	0.68 (-8.08, 10.28)	0.889
	Site 2	-0.087 (-0.169, -0.005)	-8.37 (-15.64, -0.47)	0.088
	Site 3	**-0.113** (-0.199, -0.028)	-10.70 (-18.12, -2.62)	0.046
	Site 4	-0.057 (-0.148, 0.035)	-5.50 (-13.87, 3.70)	0.281
	Site 5	**-0.203** (-0.312, -0.093)	-18.36 (-26.90, -8.81)	0.013
	Site 6	-0.043 (-0.145, 0.060)	-4.21 (-13.65, 6.25)	0.450
Number of items sold in non-intervention categories		**0.0004** (0.0002, 0.0005)	0.04 (0.02, 0.05)	0.003
Number of days pre- or post-intervention[2]		-0.0002 (-0.0009, -0.0005)	-0.02 (-0.09, 0.05)	0.587
Day of the week (Ref = Monday)	Tuesday	-0.023 (-0.068, 0.023)	-2.26 (-6.66, 2.35)	0.373
	Wednesday	-0.011 (-0.057, 0.035)	-1.10 (-5.59, 3.59)	0.650
	Thursday	**0.067** (0.018, 0.114)	6.90 (1.87, 12.19)	0.038
	Friday	0.056 (0.004, 0.106)	5.76 (0.44, 11.36)	0.082
Price increase		-0.05 (-0.127, 0.026)	-4.91 (-11.99, 2.72)	0.252
Site-supplied free lunches		**-1.78** (-1.95, -1.62)	-83.19 (-85.75, -80.17)	<0.0001
Intercept		**11.10**		<0.0001

[1] As the p-values and CIs presented here have been calculated using different assumptions (it is not possible to calculate 95% CIs that correspond to the more robust Kenward-Roger adjusted p-values), the 95%CIs may cross zero while the p-values are not significant.

[2] Day 0 is the day that the intervention was implemented

Coefficients in bold are significant at p<0.05.
