# Supplementary material for: Impact of increasing the proportion of healthier foods available on energy purchased in worksite cafeterias: A stepped wedge randomized controlled pilot trial
Source: Appetite. 2019 Feb 1;133:286–96. doi: 10.1016/j.appet.2018.11.013 (PMC6335439; doi:10.1016/j.appet.2018.11.013)
Supplement: s3_V2 [file mmc3.pdf]

S3 Table. Regression coefficients for availability intervention variables in analyses with total energy purchased daily as the outcome

Analysis	Variable		Coefficients (95% CIs)[1]	Percentage change (95% CIs)[1]	p[1]
Overall	Availability intervention period		**-0.075 (-0.119,-0.031)**	-7.19 (-11.18,-3.03)	0.021
By site	Availability intervention period	Site 1	-0.007 (-0.082,0.068)	-0.66 (-7.84,7.07)	0.870
		Site 2	-0.081 (-0.148,-0.014)	-7.78 (-13.75,-1.37)	0.088
		Site 3	**-0.099 (-0.170,-0.029)**	-9.46 (-15.66,-2.84)	0.046
		Site 4	-0.073 (-0.149,0.002)	-7.06 (-13.84,0.23)	0.281
		Site 5	**-0.213 (-0.303,-0.123)**	-19.17 (-26.11,-11.56)	0.013
		Site 6	-0.047 (-0.131,0.038)	-4.55 (-12.29,3.87)	0.450

[1] As the p-values and CIs presented here have been calculated using different assumptions (it is not possible to calculate 95% CIs that correspond to the more robust Kenward-Roger adjusted p-values), the 95%CIs may cross zero while the p-values are not significant.

Coefficients in bold are significant at p<0.05.
