# Supplementary material for: Impact of increasing the proportion of healthier foods available on energy purchased in worksite cafeterias: A stepped wedge randomized controlled pilot trial
Source: Appetite. 2019 Feb 1;133:286–96. doi: 10.1016/j.appet.2018.11.013 (PMC6335439; doi:10.1016/j.appet.2018.11.013)
Supplement: s4_V2 [file mmc4.pdf]

S4 Table. Regression coefficients for availability intervention variables in analyses with square-root total daily revenue (GBP) as the outcome

**Analysis**	**Variable**		**Coefficients (95% CIs)[1]**	**p[1]**
Overall	Availability intervention period		0.14 (-0.19, 0.48)	0.454
By site	Availability intervention period	Site 2	-0.24 (-0.73, 0.26)	0.409
		Site 3	-0.19 (-0.69, 0.31)	0.497
		Site 4	0.36 (-0.17, 0.89)	0.259
		Site 5	0.56 (-0.09, 1.19)	0.161
		Site 6	0.78 (0.18, 1.38)	0.063

[1] Coefficients are based on the square-root transformed analysis. As the p-values and CIs presented here have been calculated using different assumptions (it is not possible to calculate 95% CIs that correspond to the more robust Kenward-Roger adjusted p-values), the 95%CIs may cross zero while the p-values are not significant.

Coefficients in bold are significant at p<0.05.
